# Excess visceral adiposity induces alterations in mitochondrial function and energy metabolism in esophageal adenocarcinoma

**DOI:** 10.1186/1471-2407-14-907

**Published:** 2014-12-03

**Authors:** Niamh Lynam-Lennon, Ruth Connaughton, Eibhlin Carr, Ann-Marie Mongan, Naoimh J O’Farrell, Richard K Porter, Lorraine Brennan, Graham P Pidgeon, Joanne Lysaght, John V Reynolds, Jacintha O’Sullivan

**Affiliations:** Department of Surgery, Institute of Molecular Medicine, Trinity College Dublin, Dublin, Ireland; Nutrigenomics Research Group, University College Dublin, Dublin, Ireland; Institute of Food and Health, University College Dublin, Dublin, Ireland; School of Biochemistry and Immunology, Trinity Biomedical Sciences Institute (TBSI), Trinity College Dublin, Dublin, Ireland

**Keywords:** Obesity, Mitochondrial dysfunction, Bioenergetics, Metabolomics

## Abstract

**Background:**

Visceral obesity has a strong association with both the incidence and mortality of esophageal adenocarcinoma (EAC). Alterations in mitochondrial function and energy metabolism is an emerging hallmark of cancer, however, the potential role that obesity plays in driving these alterations in EAC is currently unknown.

**Methods:**

Adipose conditioned media (ACM) was prepared from visceral adipose tissue taken from computed tomography-determined viscerally-obese and non-obese EAC patients. Mitochondrial function in EAC cell lines was assessed using fluorescent probes, mitochondrial gene expression was assessed using qPCR-based gene arrays and intracellular ATP levels were determined using a luminescence-based kit. Glycolysis and oxidative phosphophorylation was measured using Seahorse XF technology and metabolomic analysis was performed using ^1^H NMR. Expression of metabolic markers was assessed in EAC tumor biopsies by qPCR.

**Results:**

ACM from obese EAC patients significantly increased mitochondrial mass and mitochondrial membrane potential in EAC cells, which was significantly associated with visceral fat area, and was coupled with a significant decrease in reactive oxygen species. This mitochondrial dysfunction was accompanied by altered expression of 19 mitochondrial-associated genes and significantly reduced intracellular ATP levels. ACM from obese EAC patients induced a metabolic shift to glycolysis in EAC cells, which was coupled with significantly increased sensitivity to the glycolytic inhibitor 2-deoxyglucose. Metabolomic profiling demonstrated an altered glycolysis and amino acid-related signature in ACM from obese patients. In EAC tumors, expression of the glycolytic marker *PKM2* was significantly positively associated with obesity.

**Conclusion:**

This study demonstrates for the first time that ACM from viscerally-obese EAC patients elicits an altered metabolic profile and can drive mitochondrial dysfunction and altered energy metabolism in EAC cells *in vitro. In vivo*, in EAC patient tumors, expression of the glycolytic enzyme *PKM2* is positively associated with obesity.

## Background

Esophageal adenocarcinoma (EAC) is an aggressive disease with overall 5-year survival rates of less than 15%, and approximately 40% for patients treated with curative intent [1]. In recent decades, the incidence of EAC has been increasing markedly in Europe, the US and Australia, and it now represents the predominant subtype [2, 3]. The increase in incidence of EAC in the West, parallels the exponential rise in obesity, which has reached epidemic proportions globally [4]. Obesity, specifically visceral obesity, is now recognized as a major risk factor for EAC [5, 6] and is also associated with increased mortality rates [7].

Visceral adipose tissue is a multi-functional organ with endocrine, metabolic and immunological functions and is demonstrated to have enhanced pro-inflammatory and pro-tumorigenic properties, when compared to subcutaneous fat depots [8]. Adipose tissue secretes a variety of adipokines and cytokines, which mediate biological effects on metabolism and inflammation [9]. Alterations in the levels of these secreted factors has been implicated in the causal relationship between visceral obesity and tumorigenesis [10], with an imbalance thought to induce a pro-tumorigenic environment [11]. However, the exact molecular mechanism(s) by which visceral obesity promotes initiation and progression of EAC remains poorly understood.

Adipose tissue is involved in the regulation of energy homeostasis, and whilst the aetiology of obesity is multifactorial the fundamental cause is energy imbalance. At the cellular level, the mitochondria play a central role in energy metabolism, accounting for ~95% of cellular energy in the form of ATP production. Mitochondria are functionally altered in tumors [12] and are involved in metabolic reprogramming known as the "Warburg effect’, which describes the shift of cancer cells from oxidative phosphorylation to glycolysis [13]. This metabolic shift facilitates rapidly proliferating cells and is implicated in both the initiation and progression of cancer [14]. In addition, multiple hallmarks of cancer, including evasion of apoptosis, unlimited proliferative potential and invasion have been linked directly or indirectly with mitochondrial alterations [12], highlighting alterations in mitochondrial function and energy metabolism as a potential mechanism by which obesity may promote tumorigenesis.

In this study, using a newly established computed tomography-determined visceral fat area (VFA) cut-off for obesity in EAC patients [15] and body mass index (BMI), we investigated the role of excess visceral adipose tissue in driving mitochondrial dysfunction and altered energy metabolism in EAC.

## Methods

### Patient recruitment and anthropometry

### Adipose tissue patient cohort

Following ethical approval (Joint St James’s Hospital/AMNCH ethical review board) and written informed patient consent, visceral adipose tissue was taken from EAC patients at the time of surgical resection. Excluded from the study were individuals who were pregnant, HIV or Hepatitis C positive or had diagnosed metastatic disease, or a history of cancer in the previous 3 years. All patients had a pre-operative diagnostic computed tomography (CT) scan, using either a Siemens Emotion single slice or a multi-slice Somatom Sensation scanner (Siemens, Erlangen, Germany), with individual scans analyzed on a Siemens Leonardo workstation (Siemens). VFA was calculated by an experienced radiologist. The cross-sectional surface area of the visceral fat compartment at the level of the inter-vertebral disc between L3 and L4 was calculated, using a previously standardised and validated technique [16]. Briefly, visceral compartments were delineated and then an automatic algorithm and a Hounsfield threshold value of -50 to -150 was used to determine the cross-sectional fat content within that area (cm^2^). Visceral obesity is defined as having a VFA exceeding 160 cm^2^ in males and 80 cm^2^ in females [15]. Patient characteristics are outlined in Table 1.

**Table 1:** **Patient ACM cohort characteristics**

	Mitochondrial function/energy metabolism study	Metabolomics study
***n***	10	39
**Diagnosis**	EAC	EAC
**Age at surgery, mean (range)**	62 (51–71)	63 (43–85)
**Gender (M:F)**	7:3	37:2
**VFA (cm** ^**2**^ **) Non-obese, mean (range)**	93 (48–145)	96 (27–153)
**VFA (cm** ^**2**^ **) Obese, mean (range)**	217 (182–258)	253 (166–384)

VFA, Visceral fat area.

### EAC tumor biopsy patient cohort

Following ethical approval (Joint St James’s Hospital/AMNCH ethical review board) and written informed consent, diagnostic biopsy specimens were taken from patients with a diagnosis of operable EAC, by a qualified endoscopist. Immediately adjacent tissue was taken for histologic confirmation, which was performed using routine hematoxylin and eosin staining. Specimens were immediately placed in RNA later (Ambion) and refrigerated for 24 h, before removal of RNA later and storage at -80°C. Anthropometric data were measured at the time of diagnosis by a single observer. Weight was measured to the nearest 0.1 kg. Height was measured to the nearest 0.5 cm. Body mass index (BMI) was calculated as weight/height^2^. Patient characteristics are outlined in Table 2.

**Table 2:** **EAC tumor biopsy patient cohort characteristics**

EAC tumor biopsy study	
***n***	29
**Diagnosis**	EAC
**Age at surgery, mean (range)**	61 (37–75)
**Gender (M:F)**	24:5
**Non-obese (BMI <30)** ***n***	22
BMI (Kg/m^2^) mean (range)	26.3 (20.5-29.8)
**Obese (BMI ≥ 30)** ***n***	7
BMI (Kg/m^2^), mean (range)	33.5 (30.2-40.5)
**Clinical TNM Stage**	
0	0
I	1
IIa	8
IIb	4
III	16
IV	0
**Nodal Status**	
N0	8
N1	21

BMI, Body mass index; TNM, Tumor-node-metastasis clinical staging classification; N0, indicates lymph node metastasis negative; N1, lymph node metastasis positive.

### Adipose conditioned media (ACM)

Visceral adipose tissue specimens were excised at the beginning of the surgical resection procedure and immediately placed in sterile transport buffer (PBS, glucose (0.1%), gentamycin (0.05 mg/mL)) prior to processing. To generate ACM, an adapted protocol from Fried and Moustaid-Moussa [17] was used. Briefly, visceral adipose tissue was finely minced, washed in PBS to remove excess blood and cultured in M199 medium (containing 0.05 mg/mL gentamycin) at a ratio of 5 g adipose tissue to 10 mL of media for 72 h. ACM was then removed and stored at -80°C until required.

### Cells and cell culture

OE33 esophageal adenocarcinoma cells were obtained from the ATCC and cultured as monolayers in Roswell Park Memorial Institute (RPMI) 1640 medium, supplemented with 10% (v/v) heat-inactivated foetal bovine serum and 1% (v/v) penicillin-streptomycin (50 U/mL penicillin, 50 U/mL streptomycin). Cells were maintained at 37°C in 95% humidified air containing 5% CO_2_.

### Crystal violet assay

Cells were fixed with 1% gluteraldehyde (Sigma-Aldrich) for 20 min at room temperature. The fixative was removed and cells were stained with crystal violet (0.1% in PBS) for 30 min at room temperature. The staining solution was then removed and cells were washed with H_2_O and allowed to air dry. Cells were incubated with Triton X (1% in PBS) on a shaker for 15 min at room temperature. Absorbance was read at 550 nm on a Wallac Victor^2^ 1420 multi-label counter.

### Functional assays

Reactive oxygen species (ROS), mitochondrial mass and mitochondrial membrane potential (ΔΨ_m_) were measured using 2,7-dichlorofluorescein diacetate (5 μM, Invitrogen), MitoTracker Green FM (0.3 μM, Invitrogen) and Rhodamine 123 (5 μM, Sigma) fluorescent probes, respectively. Cells were seeded in 96-well plates at a density of 5,000 cells/well, allowed to adhere overnight and incubated with either ACM or M199 media (100 μL) for 24 h. Cells were washed with a buffer containing 130 mM NaCl, 5 mM KCl, 1 mM Na_2_HPO_4_, 1 mM CaCl_2_, 1 mM MgCl_2_ and 25 mM Hepes, (pH 7.4) and then incubated with either 2, 7-dichlorofluorescein diacetate, MitoTracker Green FM or Rhodamine 123 in buffer for 30 min at 37°C. Staining buffer was removed and cells were washed with buffer. Fluorescence was measured at an excitation of 485 nm and an emission of 525 nm using a Wallac Victor^2^ 1420 multi-label counter (Perkin Elmer). Fluorescence values were normalized to cell numbers using the crystal violet assay.

### RNA isolation

Cells were seeded in 6-well plates at a density of 500,000 cells/well, allowed to adhere overnight and incubated with ACM or M199 media for 24 h. Total RNA was then isolated from cells using TriReagent® RNA isolation reagent (Molecular Research Centre Inc.) as per the manufacturer’s instructions. RNA from patient tumor tissue samples was isolated using an All-in-One purification kit (Norgen Biotek). RNA was quantified spectrophotometrically using a Nanodrop 1000 spectrophotometer v3.3 (Thermo Scientific).

### Mitochondrial arrays

RNA (1 μg) was reversed transcribed to cDNA using a First Strand cDNA synthesis kit (Qiagen), as per the manufacturer’s instructions. cDNA samples were applied to RT^2^Profiler™ PCR Arrays (Qiagen), and qPCR was performed as per the manufacturer’s instructions using an ABI Prism 7900HT real-time thermal cycler (Applied Biosystems). The averaged expression of *B2M* and *GAPDH* was used as an endogenous control for data normalisation. Data was analysed by the 2^-ΔΔCt^ method using SDS RQ 1.2 relative quantification software (Applied Biosystems). One sample was set as the calibrator for the analysis.

### Intracellular ATP measurement

Cells were seeded at a density of 10,000 cells/well in 96-well white-walled plates and allowed to adhere overnight. Relative intracellular ATP levels were measured using the luminescence-based ATPLite™ assay system (Perkin Elmer), as per the manufacturer’s instructions. Luminescence was measured using a Wallac Victor^2^ 1420 multilabel counter. An additional plate was set up concurrently and a crystal violet assay was performed to normalise ATP measurements to cell number.

### OCR and ECAR measurements

Oxygen consumption rates (OCR) and extracellular acidification rates (ECAR) were measured before and after treatment with 2-deoxyglucose (2-DG) (55 mM, Sigma), using a Seahorse XF24 analyzer (Seahorse Biosciences). Briefly, OE33 cells were seeded at 12,000 cells/well in a 24-well cell culture XF microplate (Seahorse Biosciences), allowed to adhere overnight and treated with either ACM or M199 media alone (100 μL) for 24 h. Cells were then washed with assay medium (unbuffered DMEM supplemented with 5 mM glucose, pH 7.4) before incubation with assay medium (0.5 mL) for 1 h at 37°C in a CO_2-_free incubator. Four baseline OCR and ECAR measurements were taken over 28 min, before injection of 2-DG. Two OCR and ECAR measurements were taken over 14 min following injection of 2-DG. All measurements were normalized to cell number using the crystal violet assay.

### *ATP5B*and *PKM2*gene expression analysis in tumor samples

RNA (0.5 μg) was reversed transcribed to cDNA using random hexamers (Invitrogen) and bioscript enzyme (Bioline). qPCR was performed using TaqMan primer probes and an ABI Prism 7900HT real-time thermal cycler (Applied Biosystems). 18S was used as an endogenous control for data normalisation. Data was analysed by the 2^-ΔΔCt^ method using SDS RQ 1.2 relative quantification software (Applied Biosystems). One sample was set as the calibrator for the analysis.

### Metabolomics

ACM samples (300 μL) were prepared by the addition of 250 μL deuterium oxide and 10 μL sodium trimethyl [2,2,3,3-^2^H_4_] propionate (TSP) (0.005 g/mL). ^1^H NMR spectra were acquired on a 600-MHz Varian NMR spectrometer (Varian Limited, Oxford, United Kingdom) by using the first increment of Nuclear overhauser effect spectroscopy pulse sequence at 25°C. Spectra were acquired with 16384 data points and 256 scans. Water suppression was achieved during the relaxation delay (2.5 s) and the mixing time (100 ms). All spectra were referenced to TSP at 0.0 ppm. ^1^H NMR ACM spectra were processed manually with Chenomx software (version 7.5; Chenomx Edmonton, Canada) and were phase and baseline corrected. Spectra were converted into 8000 spectral regions of 0.001 ppm width. The water region was excluded (4–6 ppm), and data were normalized to the total area of the spectral integral. Discriminating metabolites were identified using libraries of pure metabolites developed in house and the Chenomx database library. Metabolites of interest were semi-quantified using Chenomx.

### Statistics

Significance was determined by two-tailed Student’s *t*-test for normally distributed data or linear regression. For metabolomic analysis, multivariate data analysis was performed with Simca-P+ software (version 12.0). Data sets were scaled using Pareto scaling. Principal component analysis (PCA) an un-supervised method, was applied to data sets to explore any overall trends in the data. Partial least square discriminant analysis (PLS-DA), a supervised technique, identifies separation between groups and was performed in order to maximise separation between variables.

The variable importance in the projection (VIP) value of each variable was examined for the PLS-DA models. The VIP values reflect the overall contribution of each variable to the PLS-DA model. All statistical analyses were performed using SPSS v18 (SPSS software Inc) or GraphPad InStat v3 (GraphPad software Inc). For all statistical analysis, differences were considered to be statistically significant at *p* < 0.05.

## Results

### ACM from viscerally-obese EAC patients induces mitochondrial dysfunction

**Figure 1:**
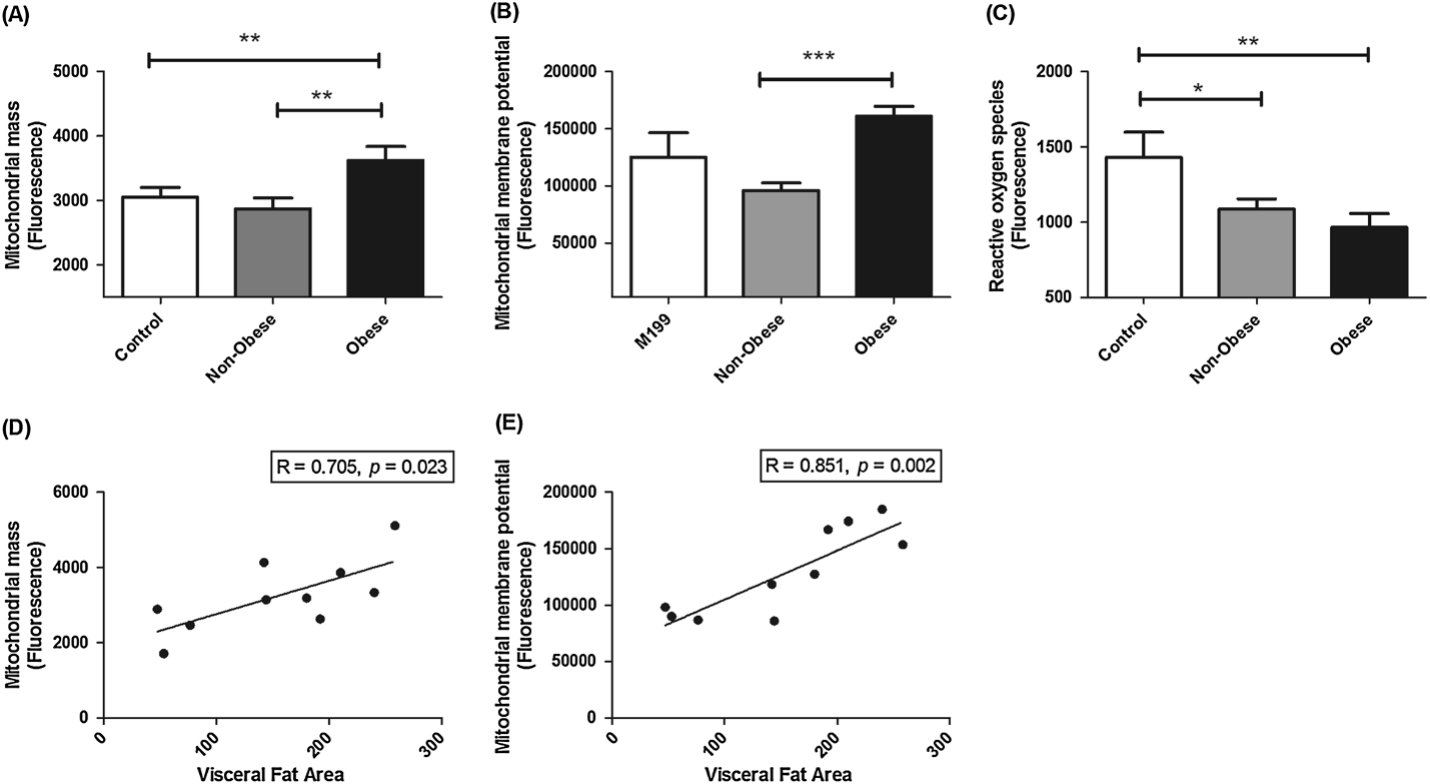
**ACM from viscerally obese EAC patients induces mitochondrial dysfunction.** OE33 cells were treated with ACM from non-obese (*n* = 5) and obese (*n* = 5) EAC patients for 24 h and mitochondrial function was assessed. Data are presented as the mean ± SEM. Analysis was performed by two-tailed Student’s *t*-test. **(A)** Mitochondrial mass was significantly increased in cells treated with ACM from obese patients, when compared to cells treated with ACM from non-obese patients and untreated controls, **p < 0.01. **(B)** Mitochondrial membrane potential was significantly increased in cells treated with ACM from obese patients, when compared to cells treated with ACM from non-obese patients, ***p < 0.001. **(C)** ROS levels were significantly reduced in cells treated with ACM from both non-obese and obese EAC patients, when compared to untreated controls, *p < 0.05, **p < 0.01. Mitochondrial Mass **(D)** and mitochondrial membrane potential **(E)** was significantly associated with visceral fat area. Analysis was performed using linear regression.

To investigate the effect of obesity on mitochondrial function in EAC, OE33 EAC cells were incubated with ACM generated from viscerally obese (*n* = 5) and non-obese EAC patients (*n* = 5) or M199 control media for 24 h. Three surrogate markers of mitochondrial function; mitochondrial mass, ΔΨ_m_ and ROS were assessed. ACM from obese patients significantly increased mitochondrial mass (*p* < 0.01) in OE33 cells (Figure 1A), when compared to M199 media alone. This effect was not demonstrated following incubation with ACM from non-obese patients. This increased mitochondrial mass was coupled with a significant increase in ΔΨ_m_ (*p* < 0.0001) in cells incubated with ACM from obese patients, when compared to non-obese patients (Figure 1B). ROS levels were significantly reduced in cells incubated with ACM from both non-obese and obese patients (Figure 1C), when compared to cells treated with M199 media. ROS levels were lower in cells treated with ACM from obese patients, when compared to ACM from non-obese patients (mean fluorescence 965 versus 1087, respectively) however, this was not statistically significant. The ACM-induced alterations in mitochondrial mass and ΔΨ_m_ were significantly positively associated with patient VFA (Figure 1D-E).

### ACM from viscerally-obese EAC patients alters the expression of mitochondrial-associated genes

**Figure 2:**
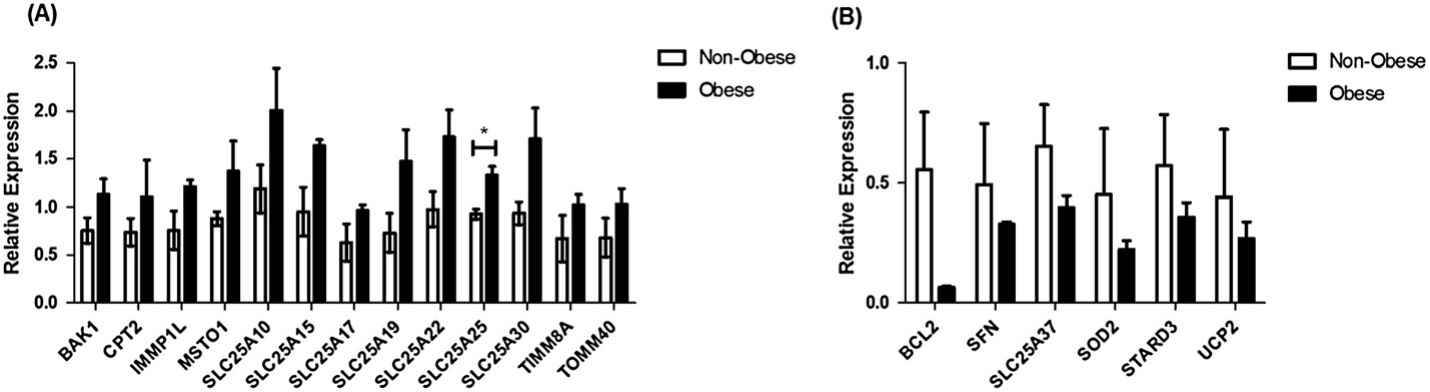
**ACM from viscerally obese EAC patients alters mitochondrial-associated gene expression.** OE33 cells were treated with ACM from non-obese (*n* = 3) and obese (*n* = 3) EAC patients for 24 h and the expression of 84 mitochondrial-associated genes was measured by qPCR-based arrays. **(A)** The expression of 13 genes was upregulated ≥ 1.5-fold following treatment with ACM from obese EAC patients, when compared to ACM from non-obese patients. Data are presented as the mean ± SEM. Analysis was performed by two-tailed Student’s *t*-test, *p < 0.05. **(B)** The expression of 6 genes was downregulated ≥ 1.5-fold following treatment with ACM from obese EAC patients, when compared to ACM from non-obese patients.

Having demonstrated ACM-induced alterations in mitochondrial function, we assessed the effect of ACM from obese (*n* = 3) and non-obese (*n* = 3) EAC patients on the expression of 84 genes involved in regulating mitochondrial function in OE33 cells. The expression of 19 genes were altered ≥ 1.5-fold following treatment with ACM from obese patients, when compared to ACM from non-obese patients. Of the altered genes, 13 were upregulated (*BAK1, CPT2, IMMP1L, MSTO1, SLC25A10, SLC25A15, SLC25A17, SLC25A19, SLC25A22, SLC25A25, SLC25A30, TIMM8A* and *TOMM40*) (Figure 2A), whilst 6 genes were downregulated (*BCL2, SFN, SLC25A37, SOD2, STARD3* and *UCP2*) (Figure 2B). ACM from obese patients significantly (*p* = 0.02) induced expression of SLC25A25 in OE33 cells, whilst the increased expression of SLC25A15 was approaching statistical significance (*p* = 0.057).

### ACM alters intracellular ATP and bioenergetics in EAC cells

**Figure 3:**
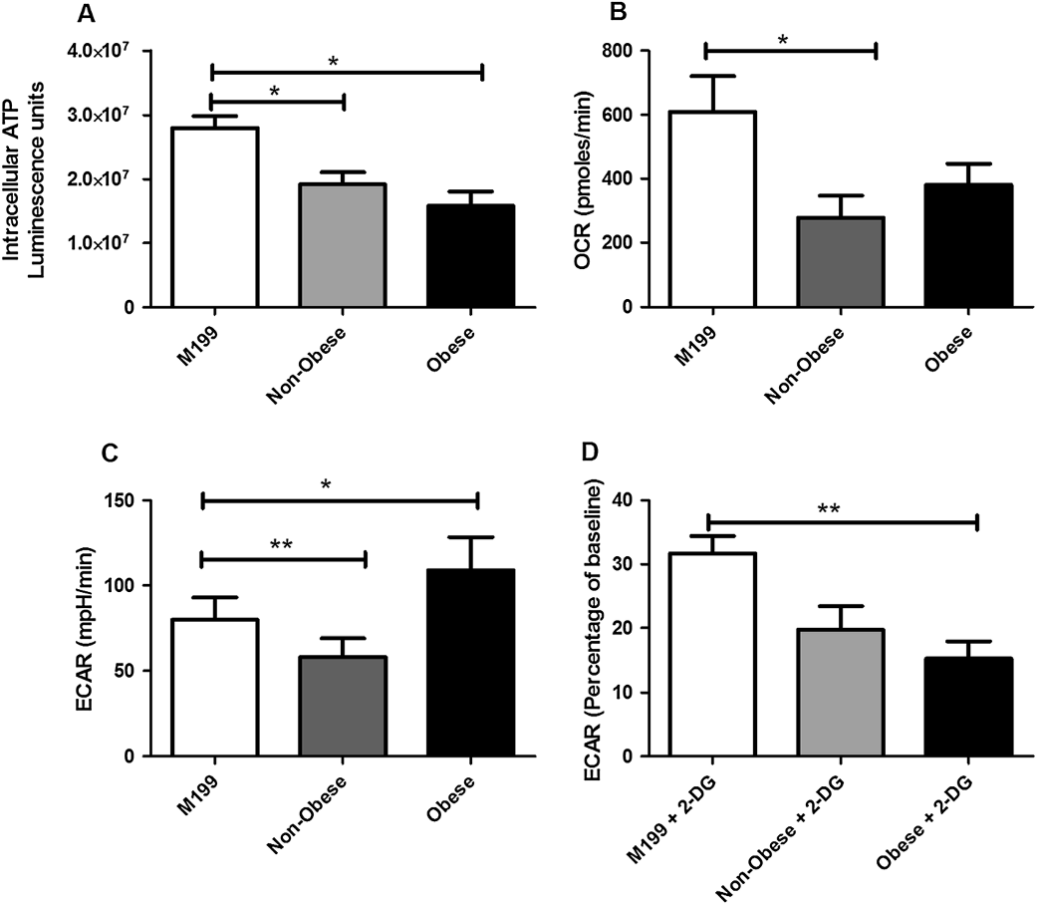
**ACM alters mitochondrial energy metabolism. (A)** Intracellular levels of ATP were significantly reduced in OE33 cells treated with ACM from both non-obese (*n* = 2) and obese (*n* = 4) EAC patients, when compared to controls. **(B)** Basal OCR was significantly reduced in OE33 cells treated with ACM from non-obese EAC patients, when compared to controls. **(C)** Basal ECAR was significantly reduced in OE33 cells incubated with ACM from non-obese EAC patients, whilst ECAR was significantly increased in OE33 cells treated with ACM from obese patients, when compared to controls. **(D)** Treatment with 2-deoxyglucose (55 mM), significantly reduced ECAR levels in OE33 cells treated with ACM from obese EAC patients, when compared to controls. Data are presented as the mean ± SEM. Analysis was performed by two-tailed Student’s *t*-test, **p < 0.01, *p < 0.05.

Given the demonstrated ACM-induced alterations in mitochondrial function and mitochondrial-associated gene expression, we investigated the effect of ACM from obese (*n* = 4) and non-obese patients (*n* = 2) on intracellular ATP levels (Figure 3A). ACM from both non-obese and obese patients significantly reduced intracellular ATP levels in OE33 cells following 24 h incubation, when compared to cells incubated with M199 media alone. To investigate if the decreased ATP levels in OE33 cells following treatment with ACM were due to alterations in energy metabolism, we measured the effect of ACM from obese and non-obese patients on two major energy pathways, oxidative phosphorylation and glycolysis in OE33 cells, using the Seahorse XF analyser. This allows the simultaneous measurement of OCR, which is a measure of oxidative phosphorylation and ECAR, a product of glycolysis, in live cells in real-time. ACM from non-obese patients significantly (*p* < 0.01) decreased OCR in OE33 cells after 24 h incubation, when compared to cells treated with M199 media alone. Whilst there was a trend towards reduced OCR in cells treated with ACM from obese patients, this was not statistically significant (*p* = 0.11). However, the effect of ACM on ECAR was starkly altered between non-obese and obese patients (Figure 3C). ACM from non-obese patients significantly decreased ECAR in OE33 cells after 24 h incubation, when compared to cells treated with M199 media. In contrast, ACM from obese patients significantly increased ECAR in OE33 cells after 24 h incubation, when compared to cells treated with M199 media alone.

To further investigate the alterations in ECAR, cells were treated with the glycolytic inhibitor 2-DG and the ECAR was assessed. OE33 cells incubated with ACM from obese patients were significantly (*p* = 0.01) more sensitive to the effects of 2-DG, when compared to cells treated with M199 media alone. In contrast, there was no significant decrease in ECAR following 2-DG treatment in cells incubated with ACM from non-obese patients, when compared to cells treated with M199 media alone.

### ACM from viscerally obese EAC patients demonstrates a significantly altered metabolic profile

**Figure 4:**
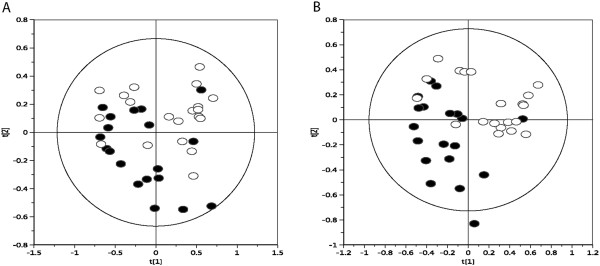
**The metabolic profile is altered in ACM from obese EAC patients. (A)** PCA plot of NMR spectra from non-obese ACM (black circles, *n* = 19) and obese ACM (white circles, *n* = 20), R^2^ = 0.672. **(B)** PLS-DA of ^1^H NMR from non-obese ACM (black circles, *n* = 19) and obese ACM (white circles, *n* = 20), R^2^ = 0.461.

To investigate what factors may be involved in the ACM-induced alterations in mitochondrial function and energy metabolism in OE33 cells, metabolomic profiling of ACM from both non-obese EAC patients was performed. To ensure adequate power for metabolomic analysis, ACM from non-obese (*n* = 19) and obese (*n* = 20) EAC patients was used. Principle component analysis revealed clear separation between ACM from non-obese and obese patients (Figure 4A). The first two components explained 67% of the variation in the data. A robust PLS-DA model (Figure 4B) was built to further explore the differences (R^2^X: 0.461, R^2^Y: 0.539, Q^2^: 0.361, Q^2^ intercept for permutation testing 0.0, -0.1). To examine the differences in metabolic signature between non-obese and obese ACM, the VIP list was obtained. The peaks with the highest scores were identified and a metabolite was assigned to each peak. Semi-quantitative concentrations were compared between non-obese and obese ACM for the most influential metabolites. Lactate was significantly increased (*p* < 0.002), whilst alanine (*p* < 0.017) and the branched-chain amino acids (BCAA) isoleucine (*p* < 0.029) and valine (*p* < 0.004) were all significantly decreased in ACM from obese patients, when compared to non-obese patients (Table 3).

**Table 3:** **Quantified metabolites normalized to standard intensity of the spectra**

% Metabolite Levels ^a^	Obese group		Non-obese group		***P***-value ^b^
	Mean	SD	Mean	SD	
Acetate	0.18	0.22	0.18	0.24	
Alanine	0.31	0.23	**0.56**	**0.37**	0.017
Ethanol	0.29	0.74	**0.34**	**0.51**	
Isoleucine	0.07	0.04	**0.12**	**0.08**	0.029
Lactate	**2.13**	**0.28**	1.75	0.62	0.023
Leucine	0.10	0.06	**0.15**	**0.10**	
Valine	0.10	0.07	**0.21**	**0.14**	0.004

^a^Metabolites that were quantified and normalized to total intensity of the NMR spectrum. ^b^ *P*-value based on independent *t*-test. Bold values indicate higher values.

### Tumor-derived expression of *PKM2*is significantly associated with obesity

Given the demonstrated ACM-induced alterations in both mitochondrial function and energy metabolism in EAC cells *in vitro*, we then investigated if metabolic alterations in EAC patient tumors were associated with obesity. We examined expression of two markers associated with energy metabolism, ATP5B a marker of oxidative phosphorylation and PKM2 a marker of glycolysis. Expression was assessed in 29 EAC tumor tissue biopsies by qPCR. As these were a retrospective cohort of patients, only BMI measurements were available. Patient obesity status was classified according to The World Health Organization (WHO) BMI guidelines [18]. Patient cohort characteristics are outlined in Table 2.

**Figure 5:**
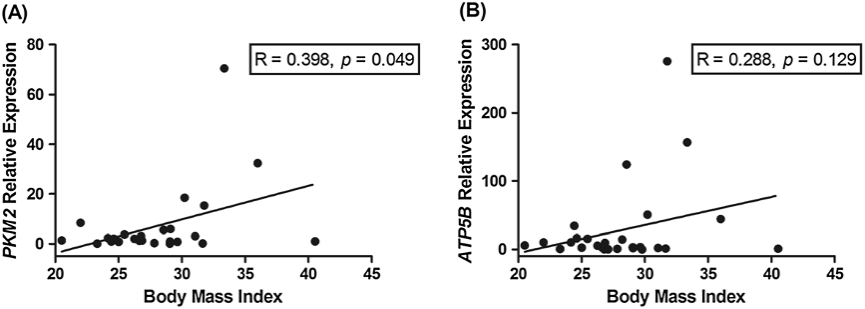
***PKM2*** **expression in EAC tumors is associated with obesity.** Gene expression profiling was performed on 29 EAC tumor biopsies by qPCR. **(A)** *PKM2* expression was significantly positively associated with BMI. **(B)** *ATP5B* expression was not associated with BMI. Analysis was performed using linear regression.

Supporting the obese ACM-induced increase in glycolysis in EAC cells demonstrated *in vitro*, expression of *PKM2* was significantly positively associated with BMI in EAC tumor biopsies (R = 0.398, *p* = 0.049, Figure 5A). There was no significant association between expression of the oxidative phosphorylation marker *ATP5B* and BMI in EAC tumor biopsies (Figure 5B). This supports our *in vitro* data and suggests that alterations in tumor energy metabolism, specifically enhanced glycolysis, is associated with obesity in EAC patients.

## Discussion

Whilst EAC has the strongest epidemiological association with obesity, the underlying molecular mechanism(s) by which obesity may drive tumorigenesis in EAC are poorly understood. Alterations in mitochondrial energy metabolism is one of the new emerging hallmarks of cancer [19]. In this study, we examined if visceral obesity drives mitochondrial dysfunction and altered energy metabolism in EAC.

Accurate assessment of obesity status is crucial for elucidating the pathophysiological role of obesity in EAC. In this study, we have used a newly established CT-determined VFA cut-off [15] for classifying visceral obesity in patients with EAC. Visceral adipose tissue has enhanced pro-tumorigenic properties, when compared to subcutaneous fat depots [8]. In this study, ACM from viscerally-obese EAC patients induced mitochondrial dysfunction in EAC cells, increasing both mitochondrial mass and ΔΨ_m_, whilst ACM from both viscerally-obese and non-obese patients significantly reduced ROS. Alterations in ΔΨ_m_ are implicated in tumorigenesis, with loss of ΔΨ_m_ associated with apoptosis and cell death [20], whilst increased ΔΨ_m_ is implicated in both cancer development and progression [21, 22]. The demonstrated obese ACM-induced increase in ΔΨ_m_ in OE33 cells may therefore suggest a mechanism by which visceral adipose tissue-derived factors promote tumorigenesis and progression in EAC. Interestingly, a study by Heerdt and colleagues [21] demonstrated that the instrinsic ΔΨ_m_ in colon cancer cells significantly correlated with invasive potential and expression of the pro-angiogenic vascular endothelial growth factor (VEGF) and the matrix metalloproteinase MMP7. Our unit has previously demonstrated that visceral adipose tissue from obese patients induces expression of another member of the matrix metallaproteinase family, MMP9, in EAC cells and that MMP9 expression in EAC tumors is significantly associated with VFA [23]. This may suggest that obese visceral adipose tissue-induced expression of markers of invasion and metastasis, such as the matrix metalloproteinase family, occurs via elevation of ΔΨ_m_.

The ACM-induced increase in mitochondrial mass and ΔΨ_m_ was significantly associated with VFA, suggesting that adipose tissue-derived factors from viscerally obese patients have enhanced paracrine effects. This is supported by two previous studies from our group, which demonstrated that ACM from viscerally-obese patients induces significantly higher proliferation and migration in EAC cells, when compared to ACM from non-obese EAC patients [8, 23]. The mitochondria are dynamic organelles, changing number, size and morphology in response to both internal and external stimuli [24]. We have previously demonstrated that levels of VEGF are significantly higher in ACM from viscerally-obese EAC patients, when compared to normal weight patients [8]. Interestingly, VEGF has been demonstrated to induce mitochondrial biogenesis [25], therefore suggesting a potential mechanism underlying the obese ACM-induced increase in mitochondrial mass in EAC cells.

Supporting the obese ACM-induced alterations in mitochondrial function, the expression of 19 genes were demonstrated to be altered in EAC cells following incubation with ACM from obese EAC patients. The majority of these altered genes were upregulated, which may be explained by the demonstrated increase in mitochondrial mass. Interestingly, the majority of genes upregulated following incubation with ACM from obese patients are involved in mitochondrial membrane transport, with *TOMM40* and *TIMM8*, which are involved in protein import into the mitochondria and 7 members of the SLC25A family of membrane transporters all upregulated, with *SLC25A25* significantly increased. The role of the SLC25A family of transporters in cancer is largely unknown. However, the expression of one member, SLC25A5, has been implicated in cancer cell metabolism, with expression shown to correlate with glycolytic metabolism in osteosarcoma and hepatocellular carcinoma cells [26]. Thus, the obese ACM-induced expression of SLC25A family members may directly alter energy metabolism in EAC cells.

Given the demonstrated ACM-induced alterations in mitochondrial function and gene expression, it is not surprising that ACM also altered both energy levels and energy metabolism in EAC cells. ACM from both obese and non-obese EAC patients reduced intracellular ATP levels in EAC cells, suggesting that adipose tissue can alter the energy state of cancer cells. ACM also altered the bioenergetics of EAC cells, with ACM from non-obese EAC patients significantly decreasing OCR, a marker of oxidative phosphorylation, and ACM from obese patients demonstrating a trend towards reduced OCR. This apparent ACM-induced reduction in mitochondrial respiration may explain the decreased ROS seen in EAC cells following incubation with ACM from both non-obese and obese patients. Decreased oxidative phosphorylation may also explain the reduction of ATP levels in EAC cells incubated with ACM, as mitochondrial respiration is the most efficient process of ATP generation [27]. This ACM-induced decrease in mitochondrial respiration in EAC cells supports previous work by Ritov *et al*. demonstrating decreased activity of the electron transport chain in skeletal muscle from obese individuals [28]. OE33 cells incubated with ACM from obese patients demonstrated increased levels of glycolysis, suggesting an ACM-induced metabolic switch to glycolysis in these cells. This glycolytic-dependence was further supported by the increased sensitivity of these cells to 2-DG -induced inhibition of glycolysis. This supports previous work demonstrating that adipose tissue from viscerally obese EAC patients induces expression of key glycolytic genes in EAC cells [29] and a study in breast cancer, which demonstrated that leptin receptor-mediated signalling was required to support aerobic glycolysis [30]. The dependence of cancer cells on glycolysis in the presence of sufficient oxygen, is probably the best known metabolic alteration in carcinogenesis [13] and is clinically exploited through the use of ^18^F-deoxyglucose-positron emission tomography [31]. Adipose tissue is an important regulator of energy homeostasis. Alterations in energy metabolism, such as glycolysis, have been demonstrated to play a key role in tumor initiation, progression and metastasis [32]. These data suggest that factors derived from visceral adipose tissue from obese EAC patients can induce a metabolic switch to glycolysis in EAC cells, suggesting a potentially important mechanism by which excess adipose tissue may promote and support tumorigenesis in EAC, and other obesity-related cancers.

The obese ACM-induced shift to glycolysis in EAC cells may seem at odds with the demonstrated increase in mitochondrial mass and ΔΨ_m_ in these cells. Whilst it was previously thought that the shift to glycolysis in cancer cells was due to an impairment of mitochondrial function [13], the demonstrated presence of functional mitochondria in numerous tumor types [33–35] has resulted in an emerging theory that suggests the glycolytic metabolic shift characteristic of cancer cells is due to enhanced glycolysis suppressing oxidative phosphorylation, rather than defects in mitochondrial respiration. The data from this study would support this hypothesis. The metabolic demand on tumor cells is greater than their non-cancer counterparts. Glycolysis, whilst less efficient that oxidative phosphorylation, makes ATP at a much faster rate [36]. Therefore, the obese ACM-induced metabolic switch to glycolysis may confer a growth/survival advantage to EAC cells.

Metabolomic analysis demonstrated an altered metabolic profile in the ACM from obese EAC patients, with significantly increased levels of the glycolytic product lactate, suggesting a shift in the flux of glycolysis towards lactate production in visceral fat from obese EAC patients. This supports previous studies which have demonstrated the production of lactate from glucose metabolism in adipocytes [37]. Lactate is now recognised to play a key role in tumorigenesis, contributing to tumor immune evasion and promoting migration of cancer cells [38], which may suggest that increased lactate secretion from visceral adipose tissue in obese EAC patients is important for driving tumor growth. Levels of alanine, and the BCAA isoleucine and valine were all significantly decreased in ACM from obese patients. Alanine can be produced by the reductive animation of pyruvate, thus this reduction in alanine may suggest a shift from pyruvate towards lactate production in visceral fat from obese EAC patients, supporting the demonstrated increase in lactate in ACM from these patients. A role for BCAA in obesity-related cancer has previously been identified, with several studies demonstrating that BCAA supplementation reduces the risk of obesity-related hepatocellular carcinoma by improving insulin resistance [39, 40]. BCAA supplementation has also been demonstrated to reduce the risk of hepatocellular carcinoma in patients with liver cirrhosis [41], decrease proliferation [42] and endothelial cell tubule formation in hepatocellular carcinoma cells, decrease neovascularisation in the liver [43], and reduce angiogenic markers such as VEGF and Tie-2 [43, 44]. Taken together, this may suggest that an imbalance in BCAAs in visceral fat from obese patients provides a mechanism for increased expression of angiogenic factors such as VEGF, which may drive growth and progression of EAC in obese patients.

Supporting the alterations in bioenergetics induced by ACM from viscerally obese patients *in vitro*, expression of the glycolytic enzyme *PKM2* in EAC tumors was significantly positively associated with BMI. This supports the glycolytic shift induced by ACM from obese patients *in vitro* and suggests that *in vivo*, a shift to glycolysis is associated with obesity and may provide a mechanism by which obesity promotes tumorigenesis in EAC. One limitation to the *in vivo* investigation in this study was the unavailability of VFA measurements. CT-determined fat area is considered the most accurate and reproducible technique of body fat measurement [45] and has been demonstrated to be a better predictor of cancer development, when compared to BMI. Therefore, future studies assessing the expression of metabolic markers in tumors from patients with VFA measurements will be vital to fully elucidate the relationship between obesity, altered energy metabolism and EAC.

## Conclusions

This study demonstrates for the first time that ACM from obese EAC patients has a distinct metabolic profile and can alter mitochondrial function, mitochondrial-associated gene expression and intracellular ATP levels in EAC cells. These alterations were accompanied by an ACM-induced metabolic remodelling, with ACM from obese EAC patients inducing a metabolic shift to glycolysis in EAC cells. This was supported *in vivo* in EAC tumors, where expression of the glycolytic marker *PKM2* was significantly positively associated with obesity. Whilst further work is required to fully elucidate the link between visceral adiposity and altered mitochondrial function and metabolism in EAC, this study suggests a novel cellular mechanism by which excess adipose tissue may promote and drive carcinogenesis in EAC.

## Abbreviations

EAC: Esophageal adenocarcinoma
VFA: Visceral fat area
CT: Computed tomography
ACM: Adipose conditioned media
ROS: Reactive oxygen species
ΔΨ_m_: Mitochondrial membrane potential
OCR: Oxygen consumption rate
ECAR: Extracellular acidification rate
2-DG: 2-deoxyglucose
TSP: Sodium trimethyl [2,2,3,3-^2^H_4_] propionate
NMR: Nuclear magnetic resonance
PCA: Principle component analysis
PLS-DA: Partial least square discriminant analysis
VIP: Variable importance in the projection
PKM2: Pyruvate Kinase M2
BCAA: Branched chain amino acids
BMI: Body mass index.
